# Identification of host plant use of adults of a long–distance migratory insect, *Mythimna separata*

**DOI:** 10.1371/journal.pone.0184116

**Published:** 2017-09-05

**Authors:** Yongqiang Liu, Xiaowei Fu, Limi Mao, Zhenlong Xing, Kongming Wu

**Affiliations:** 1 State Key Laboratory for Biology of Plant Diseases and Insect Pests, Institute of Plant Protection, Chinese Academy of Agricultural Sciences, Beijing, China; 2 Nanjing Institute of Geology and Palaeontology, Chinese Academy of Sciences, Nanjing, China; Universita degli Studi della Basilicata, ITALY

## Abstract

Adults of many insect species often become contaminated with pollen grains when feeding. Identification of plant hosts for *M*. *separata* moths could increase our understanding of their geographic origin and the coevolution of *M*. *separata* moths and their host plants. However, identifying the diet of noctuid moths using traditional direct observation is limited by their nocturnal and flight habits. In this study, we used core barcode markers and pollen morphology to identify pollen species. We found pollen from 13 plant species belonging to nine families on trapped *M*. *separata* moths, mainly from Angiosperm, Dicotyledoneae. Pollen was found on 14.4% and 12.3% of females and males, respectively, and the amount of pollen transported varied with the body part, with the most pollen on the proboscis. We were able to determine from this that the moths visited woody plants more than herbaceous plants, but not significantly so, and that they carried more pollen earlier in the migration season. In this study, we clarified the species and frequencies of pollen deposition on *M*. *separata* moths. These findings improve our understanding of the coevolution of the moths and their host plants. Identification of plant hosts for adult moths provides a new means of studying noctuid moth-host plant interactions, and informs the development of more efficient management practices for *M*. *separata*.

## Introduction

Plant and insects have co-evolved all over the world into patterns of interactions that are often mutually beneficial [1, 2]. Plants depend on insects for pollination and simultaneously provide pollen, nectar and other plant exudates for insects [1]. Understanding the interactions between plants and insects is critical to interpreting ecological and evolutionary phenomena, and the first step is to understand the range of host plants used by specific herbivorous insects.

Lepidoptera (butterflies and moths) are probably the most diverse group of phytophagous insects [3], encompassing more than 155,000 recognized species [4,5]. Noctuidae is the largest family of Lepidoptera, containing over 40,000 currently described species [6]. Noctuids are a prominent element of terrestrial ecosystems, functioning as herbivores and pollinators, as well as one of the most damaging groups of pests to agriculture. They feed on plants as larvae and on nectar as adults [7]. Although direct laboratory and field observations can provide insight into the larval diets [8–11], ascertaining the host plants of adults using these methods is subject to several compounding limitations, including their nocturnal and flight habits in the field. Therefore a new method is needed, using a combination of DNA barcoding and pollen morphology.

Adults often become contaminated with pollen during the process of nutritional supplementation, and this pollen can be identified to determine the insect’s host plants [1]. Although conventional pollen morphology is widely used for plant identification, in some cases, the pollen of closely related plants has very similar morphology [12–13]. Alternatively, DNA bar-coding has performed well for the determination of the dietary composition of various organisms [14–17]. For this purpose, a partial region of plant DNA sequence is amplified and compared to a reference database such as GenBank [14–15]. Previous studies have demonstrated that such molecular markers can potentially identify plants in the diet of insect herbivores to the family or genus level [14–15]. However, an unambiguous, reliable identification of the host plants to the species level using molecular markers is not yet possible [17]. Therefore, in the present study, DNA barcoding was used along with pollen morphology to identify the pollen species.

*Mythimna separata* is an important migratory pest in eastern Asia that undertakes a seasonal migration in China [18–20]. However, its geographic origin has still not been confirmed. The identification of pollen found on an insect’s exterior provides evidence of its origins, as some plants grow only in certain ecological zones or geographic locations. The objective of this study was to determine the host relationship and geographic origin of *M*. *separata* moths by identifying the pollen that adhered to them during their long-distance migration. We also investigated the quantities of pollen attached to various parts of the bodies of *M*. *separata* moths.

## Methods

### Ethics statement

No specific permits were required for the collection of *Mythimna separata*.

### Collection of *Mythimna separata* moths

*Mythimna separata* moths were collected using light traps. Collection was conducted every night from April to October, 2013–2015 on the 2.5 km^2^ island of Beihuang (BH, 38°24′N; 120°55′E) in the Bohai Strait, which is a major pathway for the seasonal migration of many insect species. BH is approximately 40 km from mainland China to the north and approximately 60 km from land to the south [21–23]. A vertical-pointing searchlight trap (model DK.Z.J1000B/t, 65.2 cm in diameter, 70.6 cm in height, and 30 in spread angle), equipped with a 1,000-W metal halide-lamp (model JLZ1000BT; Shanghai Yaming Lighting Co. Ltd., Shanghai, China) was suspended from the top of a house (at 500 m elevation). Twenty *M*. *separata* (or all individuals if the total captured was <20) were removed from the bags of the nylon net (60 mesh) each morning and killed by crushing their thorax, and then each moth was transferred to a 2-ml tube and stored in a freezer (-20°C) for later microscopic examination.

### Pollen preparation and SEM examination

To collect pollen from the *M*. *separata* moths, the adult’s heads were removed from the body and examined at 200× magnification using a stereomicroscope (Olympus SZX16, Pittsburgh, PA) to make a preliminary identification of the attached pollen. As in Bryant *et al*. [24], pollen was sought on only the proboscis and antenna, which were the parts that most frequently carried the pollen. To prevent contamination, a piece of paper towel (9 × 9 cm) was placed on the microscope stage and changed with each new sample, and the forceps were cleaned after each sample. The pollen grains were isolated from the proboscis or antennae, mounted on aluminum stubs, coated with gold in a sputter coater, and immediately photographed using a Hitachi S-4800 SEM (Hitachi High-Technologies Co., Tokyo, Japan).

### Analysis of DNA from the pollen grains

Single pollen grains were isolated from the aluminum stubs using a micropipette with a 3–5 μm diameter tip made by the Micropipette Puller (micropipette puller, Sutter Instruments, USA) and soaked in 1 μL of lysis solution (0.1 M NaOH)(Beijing Chemical Reagent Co. Ltd., Beijing, China) plus 2% Tween-20 (Beijing Chemical Reagent Co. Ltd.) in individual PCR tubes. The tubes were spun briefly to precipitate the pollen grains, after which 5 μL of mineral oil was added to prevent a volume loss of the solution during lysis at 95°C. The samples were spun briefly (1 min at 1,000 g), and after a 17 min and 30 s preheating treatment (95°C), the pollen grains were lysed. Equimolar aliquots of 1 μL TE buffer were added to neutralize the samples, which were spun briefly [25]. The resulting solution was used as the template for Whole Genome Amplification (WGA) using an illustra GenomiPhi V2 DNA Amplification Kit (GE Healthcare UK Ltd.). The WGA products of single pollen grains were used to amplify the plant plastid DNA.

After DNA extraction, the partial region of chloroplast *rbcL* was amplified via PCR. The nucleotide sequences (5′ to 3′) of the primers were as follows: primers *rbcla* forward (5′-ATGTCACCACAAACAGAAAC-3′) and *rbcLa* reverse (5′-TCGCATGTACCTGCAGTAGC-3′) [26]; primers *rbclb* forward (5′-ATGTCACCACAAACAGAAAC-3′) and *rbcLb* reverse (5′-GAAACGGTCTCTCCAACGCAT-3′) [27]. The PCR amplification was performed using a thermal cycler (GeneAmp PCR System 9700, Applied Biosystems, Foster City, CA) in a 50-μl reaction according to the manufacturer’s instructions. The PCR cycles consisted of an initial denaturation step for 4 min at 94°C; 35 cycles of denaturation at 94°C for 30 s, annealing at 55°C for 30 s, and extension at 72°C for 45 s; and a final extension at 72°C for 10 min. Five sequencing replicates were taken for each grain of pollen. The reaction volume of the PCR was 50 μl, which contained 100 ng of extracted DNA, 0.2 μM primer pairs, 2 mM dNTPs, 5 μl 10 × LA Taq buffer, and 1 U LA Taq polymerase (TaKaRa, Beijing, China).

Following PCR, the *rbcL* amplicons were purified using a Gel Extraction Kit (Tiangen, Beijing, China) and cloned using the pGEM-T East Vector System (Promega, Madison, WI). Each DNA-containing plasmid was isolated from cultured *E*. *coli* cells by an alkaline iniprep method. Successful insertion was verified by PCR using M_13_ forward (5′-GTTTTCCCAGTCACGAC-3′), and reverse primers (5′-CAGGAAACAGCTATGAC-3′) and Sanger sequencing was done by the Biomed company (Beijing, China).

### Pollen identification and characteristics of the pollen source plants

Identification of the *rbcL* sequences was performed individually through similarity BLAST searches against GenBank [28]. An unknown sequence was considered a member of the best hit of the query sequences when it was completely consistent with them, and the unknown species sequence was considered to be the same genus as indicated by its top hits if there were differences between the sequences. The species were corrected according to their morphological features. The pollen’s morphological features were identified using the published SEM images in the atlas of pollen flora of China and pollen flora of China woody plants by SEM [29–30]. Pollen grains that could be classified to genus or species level were used to identify the source plants of pollen.

### Data analysis

One-way ANOVA and Tukey’s Honestly Significant Difference (Tukey’s HSD) were used to evaluate differences in the frequency of pollen deposits on *M*. *separata* during different migration phases. Student’s *t*-test was used to compare differences in the annual mean frequency of pollen occurrence on female and male body parts (proboscis, antennae, or the total [combined proboscis and antennae]) of *M*. *separata* moths, and differences in the annual mean frequency of pollen deposits on the proboscis or antennae of female, male, and total (female and male) *M*. *separata* moths. A chi-square test was used to compare differences in the frequency of pollen deposits on female and male body parts (proboscis, antennae or the total [proboscis and antennae]) of *M*. *separata* moths in each year, the differences in the rates between the proboscis and the antennae of female, male, and total (female and male) *M*. *separata* moths in each year, and the characteristics of the pollen source plants. All statistical computations were performed using SPSS 13.0 (SPSS Inc., Chicago, IL, USA) [31] and all proportion data were logit transformed before being analyzed.

## Results

### Plant hosts inferred from pollen

**Fig 1:**
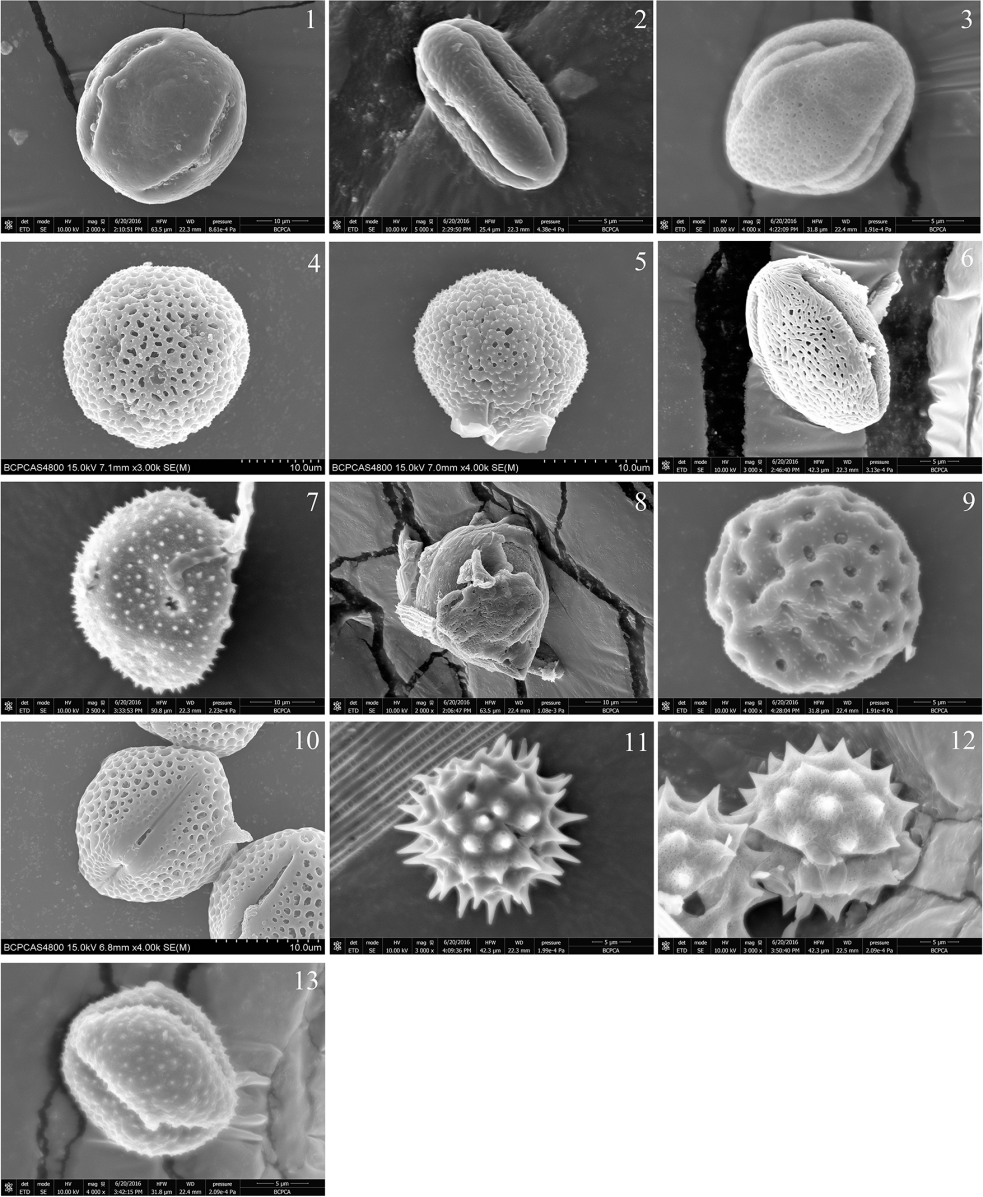
SEM microphotographs of the examined pollen species. 1. *Melia azedarach*. 2. *Castanea mollissima*. 3. *Amorpha fruticosa*. 4. Araliaceae. 5. Euphorbiaceae. 6. *Citrus L*. 7. *Adenophora trachelioides/Adenophora remotiflora*. 8. *Styphnolobium japonicum*. 9. *Chenopodium album*. 10. *Flueggea virosa*. 11. Compositae [also similar to *Aster* L.]. 12. *Aster* L. [also similar to *Chrysanthemum*, *Matricaria*]. 13. *Artemisia*.

Most of plant hosts were identified from single moths and only several moths harbored more than one plant species. Thirteen pollen-source species were identified, from nine families on *M*. *separate* moths using a combination of DNA *rbcL* data and pollen morphology. Six of the 13 pollen-source samples were identified to the species level: *Melia azedarach* Linn., *Castanea mollissima* Blume, *Amorpha fruticosa* Linn., *Styphnolobium japonicum* (L.) Schott., *Chenopodium album* L. and *Flueggea virosa* (Roxb. Ex Woigt) Voigt, and four to the genus level: *Citrus*, *Adenophora*, *Aster*, and *Artemisia* (Table 1, Fig 1). The geographic distribution of the pollen source plants is shown in Table 1 [32–33]. The success rates for a combination of pollen morphology, DNA barcoding, and distribution data identifying pollen to the species- and genus levels were 46.2 and 76.9%, respectively, while DNA barcoding alone had a success rate of 15.4 and 61.5%, and pollen morphology alone had a success rate of 7.7 and 69.2%.

**Table 1:** Molecular and morphological identification of plant species from pollen carried by *M*. *separata* and the geographic distribution of the pollen source plants.

Number	Identified plants	*rbcL*-molecular identification	Pollen morphology identification	Geographic distribution in China
1	*Melia azedarach*	*Sister to Melia azedarach*	*Melia* L.	HB_1_, SD, SX_1_, SX_2_, GS, T, HN_1_, JS, AH, ZJ, HB_2_, HN_2_, JX, SC, YN, FJ, GZ, GD, GX, HN_3_, TW, SH, CQ
2	*Castanea mollissima*	*Sister to Castanea mollissima*/*Castanea sativa*	*Castanea* Mill.*/Castanopsis* Spach.*/Lithocarpus* Bl.	HB_1_, LN, SX_1_, SX_2_, GS, T, HN_1_, JS, AH, ZJ, HB_2_, HN_2_, JX, SC, YN, FJ, GZ, GD, GX, HN_3_, TW, SH, CQ, BJ, TJ, T
3	*Amorpha fruticosa*	*Sister to Amorpha fruticosa/Amorpha canescens*	*Amorpha* L.	SX_1_, SC, CQ, JS, GZ, HN_2_, JX, GD, ZJ
4	Araliaceae	Unidentifiable	Araliaceae	All over China except XJ
5	Euphorbiaceae	Unidentifiable	Euphorbiaceae	All over China
6	*Citrus L*.	*Sister to Citrus L*.	*Rutaceae/ Rosaceae*	SD, SX_1_, SX_2_, GS, T, HN_1_, JS, AH, ZJ, HB_2_, HN_2_, JX, SC, YN, FJ, GZ, GD, GX, HN_3_, TW, SH, CQ, T
7	*Adenophora trachelioides/Adenophora remotiflora*	*Sister to Adenophora trachelioides/Adenophora remotiflora/Hanabusaya asiatica*	*Adenophora* Flash.	HLJ, JL, LN, IM, HB_1_, BJ, TJ, SX_1_, HN_1_, SD, JS, AH, HB_2_, CQ, JX, ZJ, FJ
8	*Styphnolobium japonicum*	*Sister to Styphnolobium japonicum*	*Sophora* L.	XJ, QH, SX_1_, SC, CQ, GZ, GX, HB_2_, HN_2_, AH, JS, ZJ,
9	*Chenopodium album*	*Sister to Chenopodium ficifolium/Chenopodium album*	*Chenopodium album*	All over China
10	*Flueggea virosa*	*Sister to Flueggea virosa/Flueggea neowawraea*	Euphorbiaceae	SD, SC, HB_2_, YN, GZ, HN_2_, JS, FJ, GD, GX
11	Compositae	Unidentifiable	Compositae [also similar to *Aster* L.]	All over China
12	*Aster* L.	Unidentifiable	*Aster* L. [also similar to *Chrysanthemum*, *Matricaria*]	All over China
*13*	*Artemisia*	Unidentifiable	*Artemisia*	All over China

HLJ, Heilongjiang; JL, Jilin; LN, Liaoning; IM, Inner Mongolia; HB_1_, Hebei; SD, Shandong; SX_1_, Shanxi; NX, Ningxia; SX_2_, Shanxi; GS, Gansu; QH, Qinghai; XJ, Xinjiang; T, Tibet; HN_1_, Henan; JS, Jiangsu; AH, Anhui; ZJ, Zhejiang; HB_2_, Hubei; HN_2_, Hunan; JX, Jiangxi; SC, Sichuan; YN, Yunnan; FJ, Fujian; GZ, Guizhou; GD, Guangdong; GX, Guangxi; HN_3_, Hainan; TW, Taiwan; BJ, Beijing; TJ, Tianjin; SH, Shanghai; CQ, Chongqing.

### Pollen detection rates by body part

**Fig 2:**
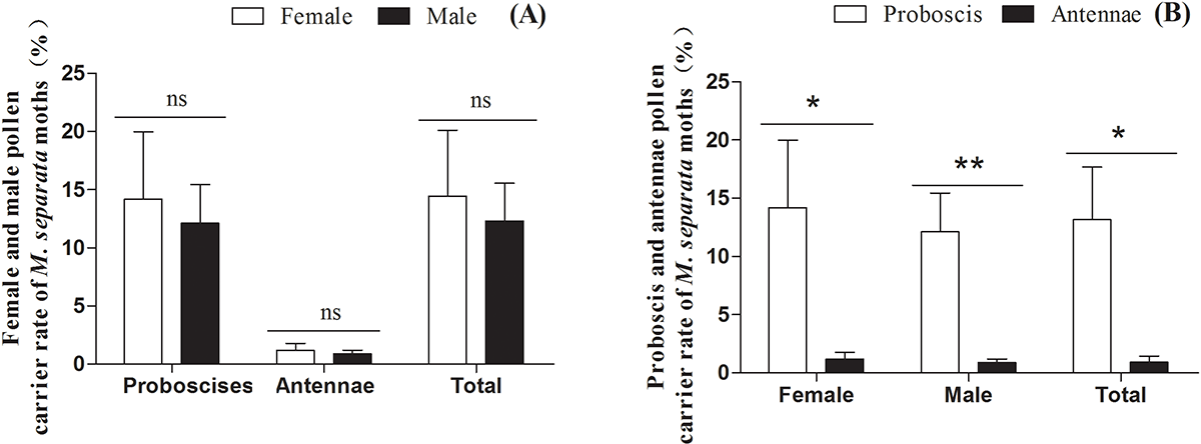
(A) Frequencies of pollen deposition on female and male proboscises, antennae and the total (proboscis and antennae) of *Mythimna separata* moths; (B) Frequencies of pollen deposition on the proboscis and antennae of female, male and total (female and male) *M*. *separata* moths. Single asterisk (*) or double asterisks (**) indicates that there was significant difference at the 1% or 5% level as determined by a Student’s t-test, and ns indicates that there was no significant difference.

No significant sex-related differences were seen in the annual mean frequency of pollen occurrence on the proboscis, antennae, or the total (combined proboscis and antennae) of *M*. *separata* moths. This was true both when years were grouped for analysis (2013–2015) (Table 2, Fig 2) (with 14.2, 1.2 and 14.4% of females and 12.1, 0.9 and 12.3% of male moths contaminated with plant pollen on the proboscis, antennae, or both of proboscis and antennae) and when the years were analyzed individually (Table 2, S1A, S1B and S1C Fig).

**Table 2:** Chi-square test and Student’s t-test for pollen deposition frequencies of *Mythimna separata*.

Female and Male Pollen Carrier Rate of *M*. *separata* Moths				Proboscis and Antennae Pollen Carrier Rate of *M*. *separata* Moths			
Proboscis	2013	χ^2^	0.474	Female	2013	χ^2^	25.94
		*df*	1			*df*	1
		*p*	0.491			*p*	<0.001
	2014	χ^2^	0.657		2014	χ^2^	13.72
		*df*	1			*df*	1
		*p*	0.418			*p*	<0.001
	2015	χ^2^	1.733		2015	χ^2^	27.94
		*df*	1			*df*	1
		*p*	0.188			*p*	<0.001
	2013–2015	*t*	0.164		2013–2015	*t*	3.908
		*df*	4			*df*	4
		*p*	0.877			*p*	0.017
Antennae	2013	χ^3^	0.054	Male	2013	χ^3^	27.77
		*df*	1			*df*	1
		*p*	0.817			*p*	<0.001
	2014	χ^3^	0.089		2014	χ^3^	14.64
		*df*	1			*df*	1
		*p*	0.765			*p*	<0.001
	2015	χ^3^	0.272		2015	χ^3^	21.81
		*df*	1			*df*	1
		*p*	0.602			*p*	<0.001
	2013–2015	*t*	0.156		2013–2015	*t*	6.386
		*df*	4			*df*	4
		*p*	0.884			*p*	0.003
Proboscis and antennae	2013	χ^3^	0.345	Female and Male	2013	χ^3^	53.68
		*df*	1			*df*	1
		*p*	0.557			*p*	<0.001
	2014	χ^3^	0.418		2014	χ^3^	29.29
		*df*	1			*df*	1
		*p*	0.518			*p*	<0.001
	2015	χ^3^	1.733		2015	χ^3^	49.57
		*df*	1			*df*	1
		*p*	0.188			*p*	<0.001
	2013–2015	*t*	0.193		2013–2015	*t*	4.243
		*df*	4			*df*	4
		*p*	0.856			*p*	0.013

The pollen detection rates were higher on the proboscis (13.2%) than on the antennae (0.9%) for 2013–2015 as a group (Table 2, Fig 2), and this difference was significant for individual years (Table 2, S1F Fig), for both female and male moths (Table 2, S1D and S1E Fig).

**Fig 3:**
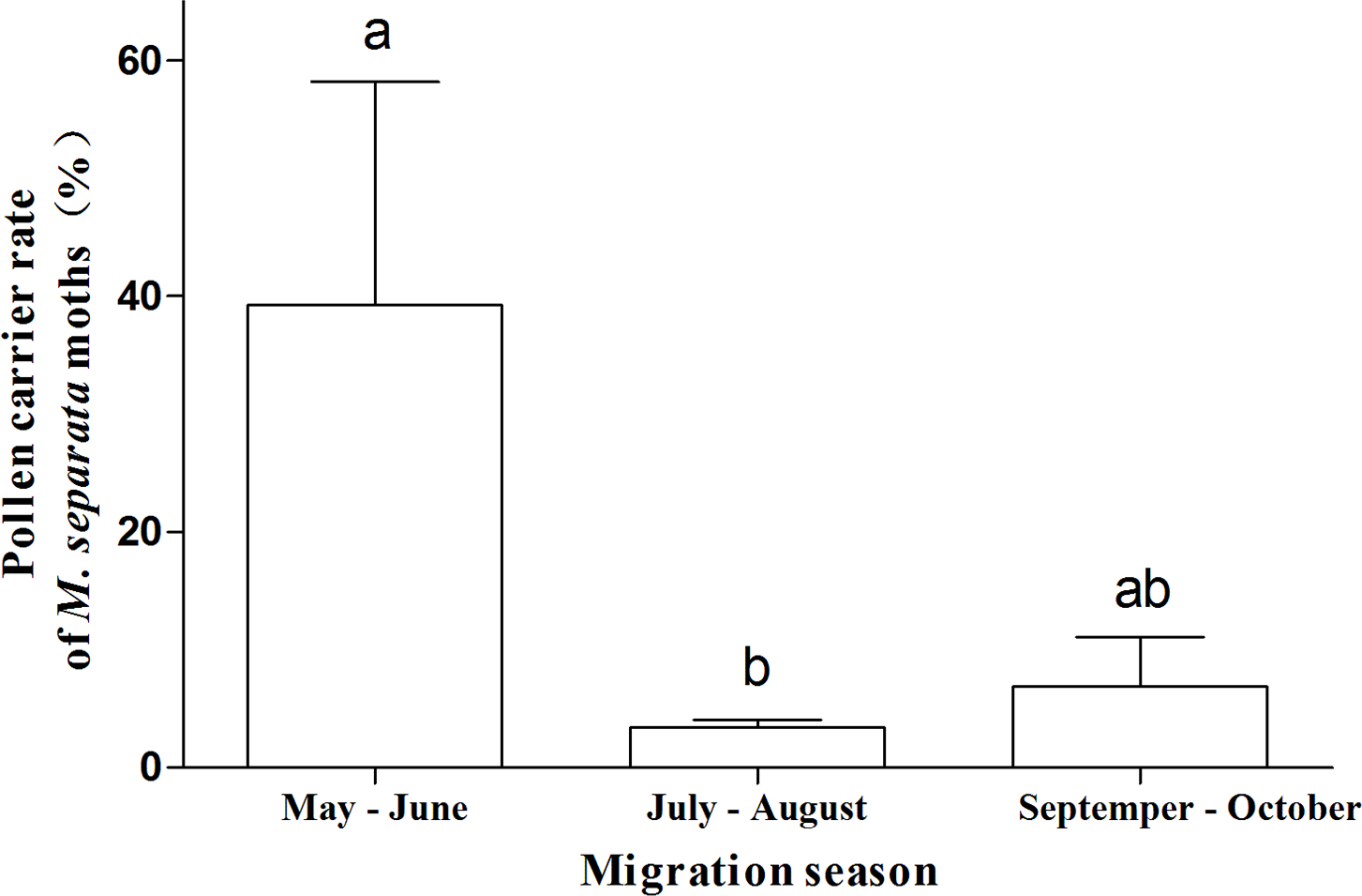
Frequencies of pollen deposition on migratory *Mythimna separata* near the Bohai Sea area in different migration stages during 2013–2015. Bars sharing the same letter mean that there were no significant differences at the 5% level by Tukey’s HSD tests.

With respect to the phenology of migration, the frequency of pollen occurrence on the bodies of *M*. *separata* was significantly higher (39.2%) in the early part of the migration period (May and June) than in the middle period (July and August) (3.4%) of the migration season, and no difference was detected between the late period (September and October) and either the early or the middle period in any of the years (*F* = 5.85, *df*_1,6_, *P* = 0.039) (Fig 3).

### Characteristics of plants whose pollen was found on *M*. *separata*

**Fig 4:**
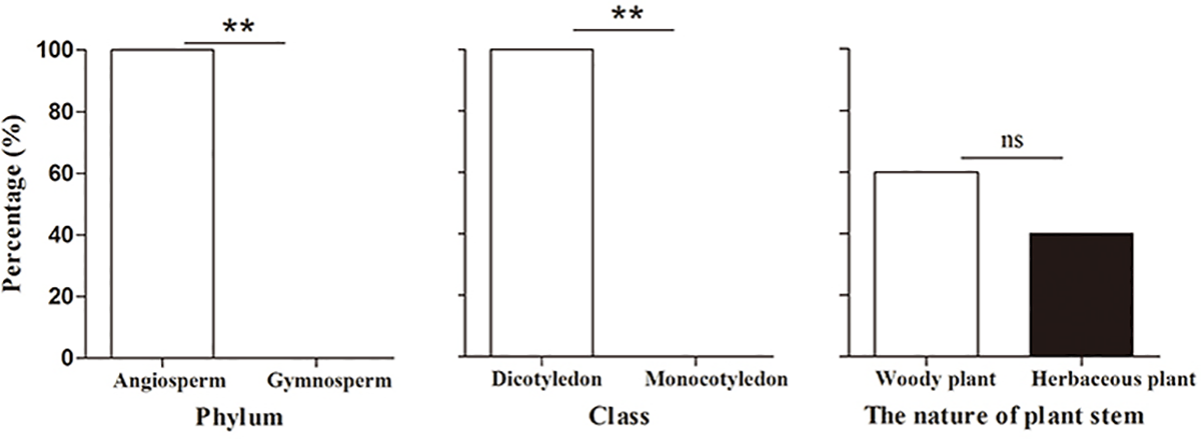
The characteristic of the pollen source plants of migratory *Mythimna separata* during 2013–2015. Single asterisk (*) or double asterisks (**) indicates there was significant difference at the 1 or 5% level as determined by a Chi-square test.

Chi-square tests showed that significantly more species whose pollen was found on *M*. *separate* moths were Angiosperm and Dicotyledon plants than Gymnosperms (*χ*^2^ = 7.50, *df* = 1, *P* = 0.006) or Monocotyledons (*χ*^2^ = 7.50, *df* = 1, *P* = 0.006). These pollen characteristics suggested that *M*. *separata* moths might have visited woody plants more often than herbaceous plants, but that difference was not significant (*χ*^2^ = 0.80, *df* = 1, *P* = 0.371) (Fig 4).

## Discussion

We used DNA bar coding, pollen morphology, and the known distributions of plants to identify the major nectar plants of *M*. *separata* moths, including 13 species from nine families that were primarily Angiosperms in the Dicotyledoneae. Our study showed that *M*. *separata* moths visited woody plants more frequently than herbaceous plants, which was a finding that was similar to that of a previous study on *Agrotis ipsilon* (Hufmagel) [34]. The rate of population increase of insects is closely related to their host plant species [35], since the quality and quantity of available food plays an important role in insect development and fecundity [36]. It is likely that noctuid moths tend to choose woody plants as a food sources, but this assumption requires further study.

The migratory behavior of some insects plays an important role in their life-history [37]. Population dynamics, capture-release-recapture experiments and radar observation have shown that *M*. *separata* is a long-distance migratory insect. The availability of nectar, which provides energy for flight, is a crucial factor that affects the duration of flight [37–38]. Host plant species for *M*. *separata* adults can be sufficiently identified by identifying the pollen species adhering to migratory *M*. *separata* because the nectar was not only collected from plants in the source area, but also from transit areas that could have been visited by the migratory *M*. *separata*.

Adult feeding is one of the most important behavioral characteristics of *M*. *separata* and has a significant influence on female fecundity [39]. Previous studies have found that when deprived of access to supplementary food, *M*. *separata* females could not regularly oviposit [40]. Although such supplementary nutrition is not directly involved in vitellogenic synthesis, it is necessary for such synthesis to be successful. In addition, the type of nutrients also affects *M*. *separata* fecundity [41], as demonstrated by Guo and Liu (1964), who found that the fecundity of *M*. *separata* moths fed *Brassica campestris* L. nectar was significantly higher than that of moths fed *Astragalus sinicus* L. nectar [42]. Whether the adult host plants identified in this study can improve the fecundity of *M*. *separata* females remains to be studied. Moreover, adult feeding has a significant effect on the flight capacity of *M*. *separata* [43], and adequate nutrition after adult emergence is necessary for successful migration [44–46]. The flight potential of *M*. *separata* adults decreases significantly after adult emergence, as migratory moths became residents within 24 h when the adults were starved [47–48]. Therefore, the pollen source plants may have a direct effect on the trans-regional migration of *M*. *separata* by providing the moths with an energy supply.

We found pollen from *M*. *azedarach*, *C*. *mollissima*, *Citrus L*., *S*. *japonicum*,and *A*. *fruticosa* on *M*. *separata* adults captured in Beihuang from late May to June. Because the Yangtze River Basin is their common distribution area [32], these adults must have visited these flowers there, and the Yangtze River Basin may thus be the natal origin of *M*. *separate* captured in Beihuang in the spring. In addition, Since the Yangtze River Basin is located in southern Shandong province, *M*. *separata* migrated to Beihuang, which is evidence for the northward spring migration of *M*. *separata*.

Traditionally, pollen identification, which is highly dependent on human expertise and vulnerable to human error, often has limited taxonomic precision and is prohibitively time-consuming for large-scale studies [49]. The novel approach of DNA-based pollen analysis has great potential for the study of plant-herbivore interactions [14]. The Consortium for the Barcode of Life-plant Working Group (CBOL) has recommended the two-locus combination of *rbcL* + matK as the best plant barcode due to its universality, sequence quality, and species discrimination [30]. Unfortunately, matK is difficult to amplify universally using currently available primer set[31]. In this study, we chose the *rbcL* intron because it has the highest level of coverage in GenBank of potential barcoding markers. The species-level identification of host plants had better have a full DNA reference library of host plant species in the target community, however, it is not possible for *M*. *separata* because of their migratory habits, so the GenBank was used in this research. *RbcL* is a good DNA barcoding region for plants at the family and genus levels, but for identification at the species level, a combination of pollen morphology and DNA barcoding is more precise. Meanwhile, other three markers (ITS, ITS2 and psbA-trnH) were recommended as primary DNA barcodes for plants at the Third International Barcoding Conference [50], and the trnL (UAA) intron chloroplast marker often used as a plant ‘DNA barcode’ for plant identification [51]. The species resolution can be increased by the combination of several barcoding [52–53], for better improvement the species discrimination, several barcoding should be used simultaneously in the future.

Adult Lepidoptera feed primarily on sugar sources [54]. We showed that some female and male moths carried pollen, which might have been contaminated during the process of nutritional supplementation. When pollen was found on the proboscis, many grains were typically present, which suggested active contact through feeding rather than casual contact through wind-blown contamination and provided evidence that the presence of nectar sources along the migration pathway of the population are a prerequisite for moths to reach their target habitat and lay eggs [55]. *Mythimna separata* carry more pollen in the early stage of the migration season, similar to *A*. *ipsilon* [37], which may be caused by differences in the abundance of nectar plants or the nutritional requirements of the moths. This topic requires further investigation.

Nectar-feeding moths are attracted to the odors of their floral hosts [56–57]. Floral volatiles play a major role in plant-insect communication [58]. The use of host volatiles has been proposed as a potential lure for both male and female insects and as a means of monitoring and forecasting populations [59–60]. We showed that *M*. *separata* adults were effective pollinators of *M*. *azedarach*, *C*. *mollissima*, *A*. *fruticosa*, *S*. *japonicum*, *C*. *album*, *F*. *virosa* and other plants. The flowers of these plants may contain specific attractant volatile components. The identification of these volatiles may allow the use of floral attractants for the management of *M*. *separata*.

Each year, *M*. *separata* undertakes a seasonal, long-distance, multigenerational roundtrip migration between southern and northern China [55]. This migration facilitates genetic exchange via pollen among plant populations across a large areas that is facilitated by adult *M*. *separata* [61]. The interactions between plants and insect herbivores are among the most important processes in terrestrial ecosystems [62–65]. Adult herbivores require nutrients or energy supplements from flowers for reproduction or flight, and flower-visiting herbivores are important pollinators [66]. The yields of many crops can be increased with the help of pollinators [67]. However, some studies confirm that the presence of adult food can enhance the herbivore population density in a range of agricultural systems [68–69]. Larvae of *M*. *separata* are a pest of millet and wheat [70] and can also occur in maize, rice and other crops [55]. Wang et al. (2006) suggested that the unprecedented increase in the geographic distribution of milk vetch from central China into south China was a key factor in the *M*. *separata* outbreaks from 1966–1977 [55]. Ultimately, more knowledge about *M*. *separate* will allow a better understanding of the regulation of the *M*. *separata* migration, allowing for the development of more efficient management practices.

## Supporting information

S1 Text The *rbcl* sequences of the pollen species investigated. (TXT)

S1 Fig Pollen deposition frequencies on the female and male proboscis (A), antennae (B) and the total (proboscis and antennae) (C) of *Mythimna separata* moths; Frequencies of pollen deposition on the proboscis and antennae of female (D), male (E) and total (female and male) (F) *M*. *separata* moths. Single asterisk (*) or double asterisks (**) indicate a significant difference at the 1% or 5% level as determined by the chi-squared test, and ns indicates no significant difference.
(TIF)

## References

[1] Jones GD, Jones SD. The uses of pollen and its implication for entomology. Neotrop Entomol 2001; 30:341–350.

[2] Qin JD, Wang CZ. The relation of interaction between insects and plants to evolution. Acta Entomol Sin 2001; 44:360–365.

[3] Scoble MJ. The Lepidoptera: Form, Function and Diversity. Oxford University Press, Oxford, 1992.

[4] Pogue MG. Lepidoptera biodiversity. *Insect Biodiversity*: *Science and Society* (ed. By Foottit R.G. and Adler P.H.), Blackwell Science Publishing, Oxford, 2009; pp. 263–293.

[5] Van Nieukerken EJ, Kaila L, Kitching IJ, Kristensen NP, Lees DC, Minet J, et al. Order Lepidoptera Linnaeus, 1758. In Zhang Z.-Q. (ed.): Animal Biodiversity: An Outline of Higher-level Classification and Survey of Taxonomic Richness. Zootaxa 2011; (3148)212-221.

[6] Wagner DL. Moths In Encyclopedia of Biodiversity; Levin S.A., Ed.; Academic Press: San Diego, CA, USA, 2001; Volume 4, pp. 249–270.

[7] Mitchell A, Mitter C, Regier JC. Systematics and evolution of the cutworm moths (Lepidoptera: Moctuidae): Evidence from two protein-coding nuclear genes. Syst. Entomol. 2006, 31, 21–46.

[8] Barone JA. Host-specificity of folivorous insects in a moist tropical forest. J Anim Ecol 1998, 67:400–409.

[9] Wood TK, Olmstead KL. Latitudinal effects on treehopper species richness (Homoptera: Membracidae). Ecol Entomol 1984; 9:109–115.

[10] Janzen DH. Ecological characterization of a Costa Rican dry forest caterpillar fauna. Biotropica 1988, 20, 120–135.

[11] Hodkinson ID, Casson D. A lesser predilection for bugs: Hemiptera (Insecta) diversity in tropical rain forests. Biol. J. Linn. Soc. 1991, 43, 101–109.

[12] Salmaki Y, Jamzad Z, Zarre S, Bräuchler C. Pollen morphology of Stachys (Lamiaceae) in Iran and its systematic implication. Flora 2008; 203:627–639.

[13] Khansari E, Zarre S, Alizadeh K, Attara F, Aghabeigic F, Salmaki Y. Pollen morphology of *Campanula* (Campanulaceae) and allied genera in Iran with special focus on its systematic implication. Flora 2012; 207:203–211.

[14] Jurado-Rivera JA, Vogler AP, Reid CA, Petitpierre E, Gómez-Zurita J. DNA barcoding insect-host plant associations. Proc R Soc Biol Sci Ser B 2009; 276:639–648. 10.1098/rspb.2008.1264 PMC2660938 19004756

[15] Navarro SP, Jurado-Rivera JA, Gomez-Zurita J, Lyal CHC, Vogler AP. DNA profiling of host-herbivore interactions in tropical forests. Ecol Entomol 2010; 35:18–32.

[16] Staudacher K, Wallinger C, Schallhart N, Traugott M. Detecting ingested plant DNA in soil-living insect larvae. Soil Biol. Biochem. 2011, 43, 346–350. doi: 10.1016/j.soilbio.2010.10.022 21317975 10.1016/j.soilbio.2010.10.022 PMC3021716

[17] García-Robledo C, Erickson DL, Staines CL, Erwin TL, Kress WJ. Tropical plant-herbivore networks: Reconstructing species interactions using DNA barcodes. PLoS ONE 2013, 8, e52967 doi: 10.1371/journal.pone.0052967 23308128 10.1371/journal.pone.0052967 PMC3540088

[18] Chen RL, Bao XZ. Research on the migration of oriental armyworm in China and a discussion of management strategy. Insect Sci Applic 1987; 8:571–572.

[19] Chen RL, Bao XZ, Drake VA, Farrow RA, Wang SY, Sun YJ, et al Radar observations of the spring migration into northeastern China of the oriental armyworm, *Mythimna separata*, and other insects. Ecol Entomol 1989; 14:149–162.

[20] Chen RL, Sun YJ, Wang SY, Zhai BP, Bao XZ. Migration of the oriental armyworm *Mythimna separata* in East Aisa in relation to weather and climate. I. Northeastern China, pp. 93–104. In Drake V. A. and Gatehouse A. G. (eds.), Insect migration: tracking resource in space and time. Cambridge University Press, Cambridge, United Kingdom, 1995.

[21] Liu YQ, Fu XW, Feng HQ, Liu ZF, Wu KM. Trans-regional Migration of *Agrotis ipsilon* (Lepidoptera: Noctuidae) in North-East Asia. Ann Entomol Soc Am 2015; 108:519–527.

[22] Feng HQ, Wu KM, Cheng DF, Guo YY. Radar observations of the autumn migration of the beet armyworm *Spodoptera exigua* (Lepidoptera: Noctuidae) and other moths in northern China. Bull Entomol Res 2003; 93:115–124. doi: 10.1079/BER2002221 12699532 10.1079/BER2002221

[23] Cheng DF, Feng HQ, Wu KM. Scanning entomological radar and radar observation for insect migration Science Press, Beijing, China, 2005.

[24] Bryant VM, Pendleton M, Murry RE, Lingren PD, Raulston JR. Techniques for studying pollen adhering to nectar-feeding corn earworm (Lepidoptera: Noctuidae) moths using scanning electron microscopy. J Econ Entomol 1991; 84:237–240.

[25] Chen PH, Pan YB, Chen RK. High-throughput procedure for single pollen grain collection and polymerase chain reaction in plants. J Integr Plant Biol 2008; 50:375–383. doi: 10.1111/j.1744-7909.2007.00624.x 18713371 10.1111/j.1744-7909.2007.00624.x

[26] Fay MF, Swensen SM, Chase MW. Taxonomic affinities of *Medusagyne oppositifolia* (Medusagynaceae). Kew Bull 1997; 52:111–120.

[27] Fazekas AJ, Burgess KS, Kesanakurti PR, Graham SW, Newmaster SG, Husband BC, et al Multiple multilocus DNA barcodes from the plastid genome discriminate plant species equally well. PLoS ONE 2008; 3:e2802 doi: 10.1371/journal.pone.0002802 18665273 10.1371/journal.pone.0002802 PMC2475660

[28] Altschul SF, Gish W, Miller W, Myers EW, Lipman DJ. Basic local alignment search tool. J Mol Biol 1990; 215:403–410. doi: 10.1016/S0022-2836(05)80360-2 2231712 10.1016/S0022-2836(05)80360-2

[29] Wang FX, Qian NF, Zhang YL, Yang HQ. Pollen flora of China. Beijing: Science Press 1995; pp. 1–461.

[30] Li TQ, Cao HJ, Kang MS, Zhang ZX, Zhao N, Zhang H, et al Pollen flora of China woody plants by SEM. Beijing: Science Press 2011; pp. 1–1233.

[31] SPSS Incorporation. SPSS 13.0 for the Windows. SPSS Inc, Chicago, IL 2006.

[32] Fang JY, Wang ZH, Tang ZY. Atlas of woody plant in China: distribution and climate Springer, Berlin, Germany, 2009.

[33] Flora of China Editorial Committee. Flora of China, Beijing: Science Press, 2005.

[34] Liu YQ, Fu XW, Mao LM, Xing ZL, Wu KM. Host plant identification for adult Agrotis ipsilon, a long-distance migratory insect. Int J Mol Sci 2016; 17:851. 10.3390/ijms17060851 PMC4926385 27271592

[35] Singh OP, Parihar SBB. Effect of different hosts on the development of *Heliothis armigera* Hub. Bull Entomol Res 1988; 29: 2168–2172.

[36] Kehat M, Wyndham M. The effect of food and water on development, longevity, and fecundity in the Rutherglen bug, *Nysius vinitor* (Hemiptera: Lygaeidae). Aust J Zool 1972; 20:119–130.

[37] Zhang ZT, Li GB. A study on the biological characteristics of the flight of the oriental armyworm [*Mythimna separata* (Walker)] moths. Acta Phytophyl Sin 1985; 12:91–100.

[38] Cao YZ, Cheng DF, Ni HX, Li GB. Effects of compensatory carbohydrate sources on the flight ability of oriental armyworm., pp. 422–427 in Anon. (Ed.) Compilation of Theses in First Science Conference of Youth Workers in Plant Protection in China. Beijing, China Science and Technology Press, 1991.

[39] Cao C. Effects of the nutrition and neuro-endocrine during adult stage on the vitellogenesis and ovary development in the orient armyworm, Mythimna separate (Walker). PhD thesis. Beijing, Beijing Agricultural University, China, 1995.

[40] Guo F. Insects Hormone. Beijing: Science Press, 1979.

[41] Guo F, Liu J L. Studies on the reproduction of the armyworm, *Leucania separata* walker (Lepidoptera: Noctuidae) П effect of supplementary nutrition on fecundity. Acta Entomol Sin 1964; 13:785–794.

[42] Zhao D. The preliminary study on attraction and nutritional effects of spring nectar source plants to oriental armyworm moth Mythimna separate. Master thesis. Zhengzhou, Henan Agricultural University, China, 2009.

[43] Zhang ZT, Li GB. A study on the biological characteristics of the flight of the oriental armyworm [*Mythimna separate* (Walker)] moth. Acta Phytophyl Sin 1985; 12:93–100.

[44] Li GB. Regularity of occurrence and control strategy in the oriental armyworm *Mythimna separata* in China, pp. 446–466. China Plant Protection Science. Science Press, Beijing, China, 1961.

[45] Li GB, Wang HX, Li SH. Study on migratory regularity and forecast of the oriental armyworm in west China. Scientia Agri. Sin. (30 anniversary of the founding of the Chinese academy of agricultural sciences, Special issue) 1987; 68–74.

[46] Wang GP, Zhang QW, Ye ZH, Luo LZ. The role of nectar plants in severe outbreaks of armyworm, *Mythimna separata* (Lepidoptera: Noctuidae) in China. Bull Entomol Res 2006; 96:445–455. 17092356

[47] Zhang L, Luo LZ, Jiang XF, Hu Y. Influences of starvation on the first day after emergence on ovarian development and flight potential in adults of the oriental armyworm, *Mythimna separata* (Walker) (Lepidoptera: Noctuidae). Acta Entomol Sin 2006; 49:895–902.

[48] Zhang L, Jiang XF, Luo LZ. Determination of sensitive stage for switching migrant oriental armyworms, *Mythimna separata* (Walker), into residents. Environ Entomol 2007; 37:1389–1395. 10.1603/0046-225x-37.6.1389 19161680

[49] Richardson RT, Lin CH, Sponsler DB, Quijia JO, Goodell K, Johnson RM. Application of ITS2 metabarcoding to determine the provenance of pollen collected by honey bees in an agroecosystem. Appl Plant Sci 2015; 3:1400066. 10.3732/apps.1400066 PMC4298230 25606352

[50] Pang X, Luo H, Sun C. Assessing the potential of candidate DNA barcodes for identifying non-flowering seed plants. Plant Biol 2012; 14: 839–844. doi: 10.1111/j.1438-8677.2011.00554.x 22309105 10.1111/j.1438-8677.2011.00554.x

[51] Jurado-Rivera JA, Vogler AP, Reid CAM, Petitpierre E, Gómez-Zurita J. DNA barcoding insect-host plant associations. P Roy Soc B 2009; 276: 639–648. 10.1098/rspb.2008.1264 PMC2660938 19004756

[52] Kress WJ, Erickson DL, Jones FA, Swenson NG, Perez R, Sanjur O, et al Plant DNA barcodes and a community phylogeny of a tropical forest dynamics plot in Panama. P Natl Acad Sci USA 2009; 106: 18621–18626. 10.1073/pnas.0909820106 PMC2763884 19841276

[53] Burgess KS, Fazekas AJ, Kesanakurti PR, Graham SW, Husband BC, Newmaster SG, et al Discriminating plant species in a local temperate flora using the *rbcL* + *matK* DNA barcode. Methods Ecol Evol 2011; 2: 333–340.

[54] Wäckers FL, Romeis J, Rijn PV. Nectar and pollen feeding by insect herbivores and implications for multitrophic interactions. Annu Rev Entomol 2007; 52:301–323. doi: 10.1146/annurev.ento.52.110405.091352 16972766 10.1146/annurev.ento.52.110405.091352

[55] Wang GP, Zhang QT, Ye ZH, Luo LZ. The role of nectar plants in severe outbreaks of armyworm *Mythimna separate* (Lepidoptera: Noctuidae) in China. Bull Entomol Res 2006; 96:445–455. 17092356

[56] Heath RR, Landolt PJ, Dueben B, Lenczewski B. Identification of floral compounds of night-blooming jessamine attractive to cabbage-looper moths. Env Entomol 1992; 21:854–859.

[57] Zhu YC, Keaster AJ, Gerhardt KO. Field observations on attractiveness of selected blooming plants to noctuid moths and electroantennogram responses of black cutworm (Lepidoptera, Noctuidae) moths to flower volatiles. Environ Entomol 1993; 22:162–166.

[58] Cunningham JP, Moore CJ, Zalucki MP, West SA. Learning, odour preference and flower foraging in moths. J Exp Biol 2004; 207:87–94. 14638836 10.1242/jeb.00733

[59] Tingle FC, Mitchell ER. Attraction of *Heliothis virescens* (F.) Lepidoptera: Noctuidae) to volatiles from extracts of cotton flowers. J Chem Ecol 1992; 18:907–914. doi: 10.1007/BF00988331 24254094 10.1007/BF00988331

[60] Udayagiri S, Mason CE. Host plant constituents as oviposition stimulants for a generalist herbivore: European corn borer. Entomol Exp Appl 1995; 76:59–65.

[61] Jiang XF, Luo LZ, Zhang L, Sappington TW, Hu Y. Regulation of migration in Mythimna separate (Walker) in China: a review integrating environmental, physiological, hormonal, genetic, and molecular factors. Environ Entomol 2011; 40:516–533. doi: 10.1603/EN10199 22251629 10.1603/EN10199

[62] Crawley MJ. Insect herbivores and plant population dynamics. Annu Rev Entomol 1989; 34:531–564.

[63] Hicke JA, Allen CD, Desai AR, Dietze MC, Hall RJ, Hogg EH, et al Effects of biotic disturbance on forest carbon cycling in the United States and Canada. Glob. Change Biol 2012; 18:7–34.

[64] Huntly N. Herbivores and the dynamics of communities and ecosystems. Annu Rev Ecol Syst 1991; 22:477–503.

[65] Schmitz OJ. Herbivory from individuals to ecosystems. Annu Rev Ecol Syst 2008; 39:133–152.

[66] Faegri K, van der Pijl L. The Principles of Pollination Ecology. Pergamon: Oxford, UK, 1979.

[67] Hendrix Ш WH, Showers WB. Tracing black cutworm and armyworm (Lepidoptera: Noctuidae) northward migration using *Pithecellobium* and *Calliandra* pollen. Environ Entomol 1992; 21:1092–1096.

[68] Prokopy RJ, Duan JJ, Vargas RI. Potential for host range expansion in *Ceratitis capitata* flies: impact of proximity of adult food to egg-laying sites. Ecol Entomol 1996; 21:295–299.

[69] Swirski E, Izhar Y, Wysoki M, Gurevitz E, Greenberg S. Integrated control of the long-tailed mealybug, *Pseudococcus longispinus* (Hom., Pseudococcidae), in adocado plantations in Israel. Entomophaga 1980; 25:415–426.

[70] Zou SW. Review of the armyworm damage and control in the historical record in China. Entomol Knowl 1956; 2:241–246.

