# Supplementary figures and images for: Identification of host plant use of adults of a long–distance migratory insect, Mythimna separata
Source: PLoS One. 2017 Sep 5;12(9):e0184116. doi: 10.1371/journal.pone.0184116 (PMC5584948; doi:10.1371/journal.pone.0184116)

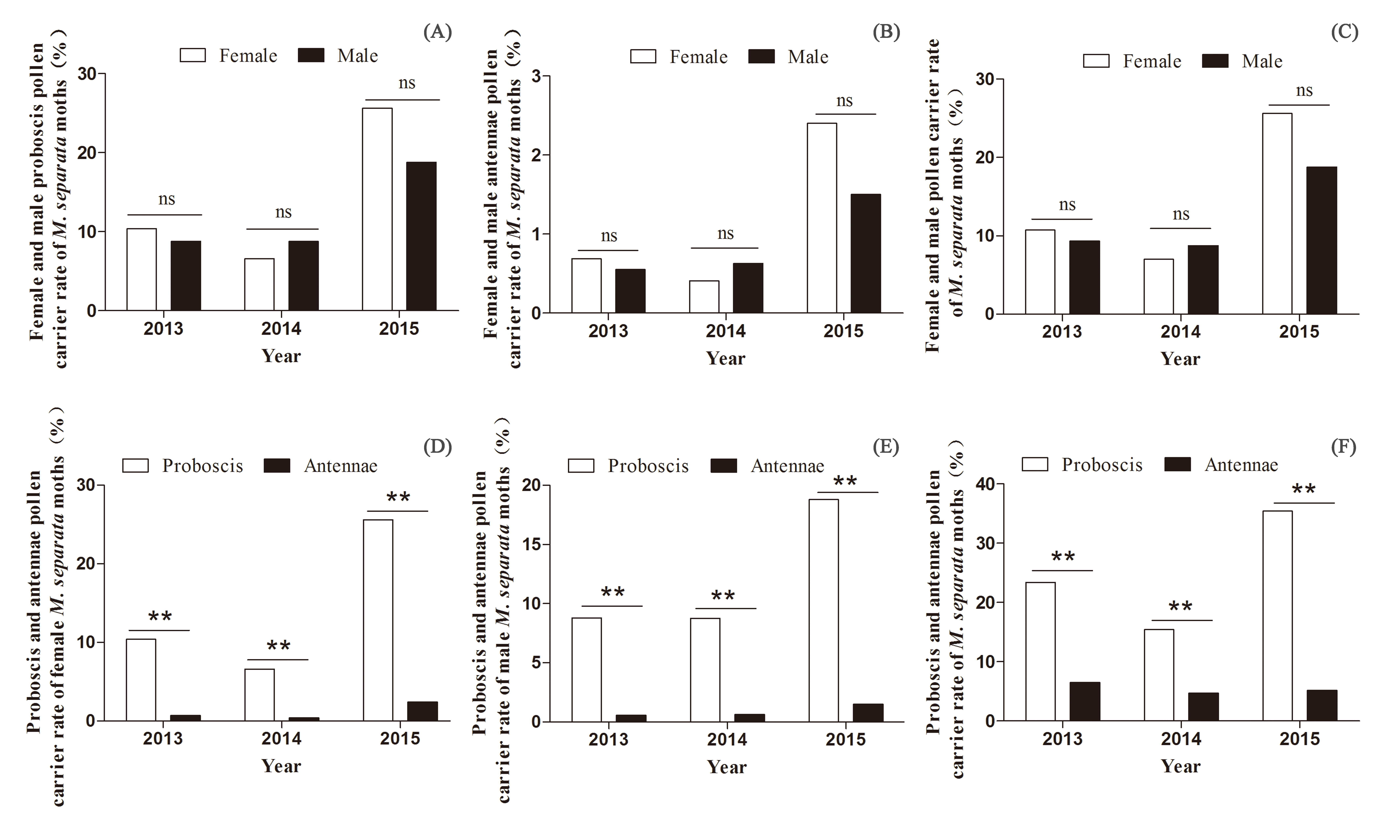

Supplement: S1 Fig — Pollen deposition frequencies on the female and male proboscis (A), antennae (B) and the total (proboscis and antennae) (C) of Mythimna separata moths; Frequencies of pollen deposition on the proboscis and antennae of female (D), male (E) and total (female and male) (F) M. separata moths. Single asterisk (*) or double asterisks (**) indicate a significant difference at the 1% or 5% level as determined by the chi-squared test, and ns indicates no significant difference. (TIF) [file pone.0184116.s002.tif]
